# Sparse coding and dictionary learning for spike trains to find spatio-temporal patterns

**DOI:** 10.1186/1471-2202-16-S1-P255

**Published:** 2015-12-18

**Authors:** Taro Tezuka

**Affiliations:** 1 Faculty of Library, Information and Media Science, University of Tsukuba, Tsukuba, 305-0821, Japan

## 

In biological neural networks, it is widely accepted that the spikes are the fundamental building blocks of information representation [1]. In contrast, whether such building blocks exist at a higher level in terms of time and in a population of neurons is a topic of ongoing debate. One approach for finding candidates for such building blocks is to seek for frequently appearing spike patterns in a population. These sequences are often called spatio-temporal patterns, cell assemblies, or unitary events [2-4]. They could metaphorically be considered as an ``alphabet'' of neural information processing [5,6]. Some patterns have already been found and are related to functional roles such as memory consolidation and gating of sensory inputs [7,8].

One difficulty in finding spatio-temporal patterns arises from observed spike trains being a superposition of multiple patterns. In signal processing, one commonly used method for decomposing the signal into patterns is dictionary learning for sparse coding [9-11]. Sparse coding expresses the input signal as a linear combination of a few template vectors taken from a matrix called a dictionary or codebook. In terms of linear algebra, sparse coding corresponds to finding a sparse vector x, which fulfills y = Dx, where y is the observed signal vector and D is a dictionary. When the dimension of × is much larger than that of y, it is possible to find sparse x. Each column of D is called an atom, which represents a template vector. A good dictionary decomposes the most of the observed signals into a small set of template vectors. In other words, D must sparsify not just one input vector y but many others as well. This is represented by using matrix Y whose column vectors are observed signals. In this case, sparse coding is represented by equation Y = DX. The goal is to find sparse matrix × given Y and D. Whether input matrix Y can be transformed into sparse × or not depends on dictionary D. The goodness of D depends on Y. The task of finding optimal D given Y is called dictionary learning. In this work sparse coding and dictionary learning were applied for finding spatio-temporal patterns from multivariate spike trains. Spike trains were transformed to vectors using binning, that is, converted to vectors of short-time firing rates. The methods were tested using different bin sizes. The results obtained for biological data showed possible candidates of spatio-temporal patterns in neural activity.

## References

[B1] Gerstner W Kistler WM Naud R Paninski L Neuronal Dynamics 2014 Cambridge: Cambridge University Press

[B2] Villa AEP Empirical evidence about temporal structure in multi-unit recordings Conceptual Advances in Brain Research 2000 3 1 51

[B3] Buzsaki G Neural syntax: cell assemblies, synapsembles, and readers Neuron 2010 68 3 362 385 21040841 10.1016/j.neuron.2010.09.023 PMC3005627

[B4] Grun S Diesmann M Aertsen A Unitary events in multiple single-neuron spiking activity. I. Detection and significance Neural Computation 2002 14 43 80 11747534 10.1162/089976602753284455

[B5] Baram Y Global attractor alphabet of neural firing modes Journal of Neurophysiology 2013 110 907 915 23678017 10.1152/jn.00107.2013

[B6] Eyherabide HG Samengo L The information transmitted by spike patterns in single neurons Journal of Physiology Paris 2010 104 147 155 10.1016/j.jphysparis.2009.11.018 19944153

[B7] Nadasdy Z Hirase H Czurko A Csicsvari J Buzsaki G Replay and time compression of recurring spike sequences in the hippocampus Journal of Neuroscience 1999 19 21 9497 9507 10531452 10.1523/JNEUROSCI.19-21-09497.1999 PMC6782894

[B8] Luczak A Bartho P Harris KD Gating of sensory input by spontaneous cortical activity Journal of Neuroscience 2013 33 4 1684 1695 23345241 10.1523/JNEUROSCI.2928-12.2013 PMC3672963

[B9] Hoyer PO Non-negative matrix factorization with sparseness constraints Journal of Machine Learning Research 2004 5 1457 1469

[B10] Bruckstein A Donoho DL Elad M From sparse solutions of systems of equations to sparse modeling of signals and images SIAM Review 2009 51 1 34 81

[B11] Elad M Sparse and Redundant Representations 2010 Berlin: Springer

